# Supplementary material for: Automatic data-driven design and 3D printing of custom ocular prostheses
Source: Nat Commun. 2024 Feb 27;15:1360. doi: 10.1038/s41467-024-45345-5 (PMC10899237; doi:10.1038/s41467-024-45345-5)
Supplement: Supplementary file 1 — Supplementary Information [file 41467_2024_45345_MOESM1_ESM.pdf]

Automatic data-driven design and 3D printing of custom ocular prostheses

Johann Reinhard[1,2], Philipp Urban[1,3], Stephen Bell[4,5], David Carpenter[6], Mandeep S. Sagoo[5]

[1]Fraunhofer Institute for Computer Graphics Research IGD, Darmstadt, Germany. [2]Department of Computer Science, Technical University Darmstadt, Darmstadt, Germany. [3]Department of Computer Science, Norwegian University of Science and Technology, Gjøvik, Norway. [4]Ocupeye Ltd., Kenilworth, UK. [5]NIHR Biomedical Research Centre for Ophthalmology at Moorfields Eye Hospital and UCL Institute of Ophthalmology, London, UK. [6]Ocular Prosthetics Department, Moorfields Eye Hospital NHS Foundation Trust, London, UK. [7]Ocular Oncology Service, Moorfields Eye Hospital NHS Foundation Trust, London, UK. [8]Retinoblastoma Service, Royal London Hospital, Barts Health NHS Trust, London, UK.

Supplementary Figures

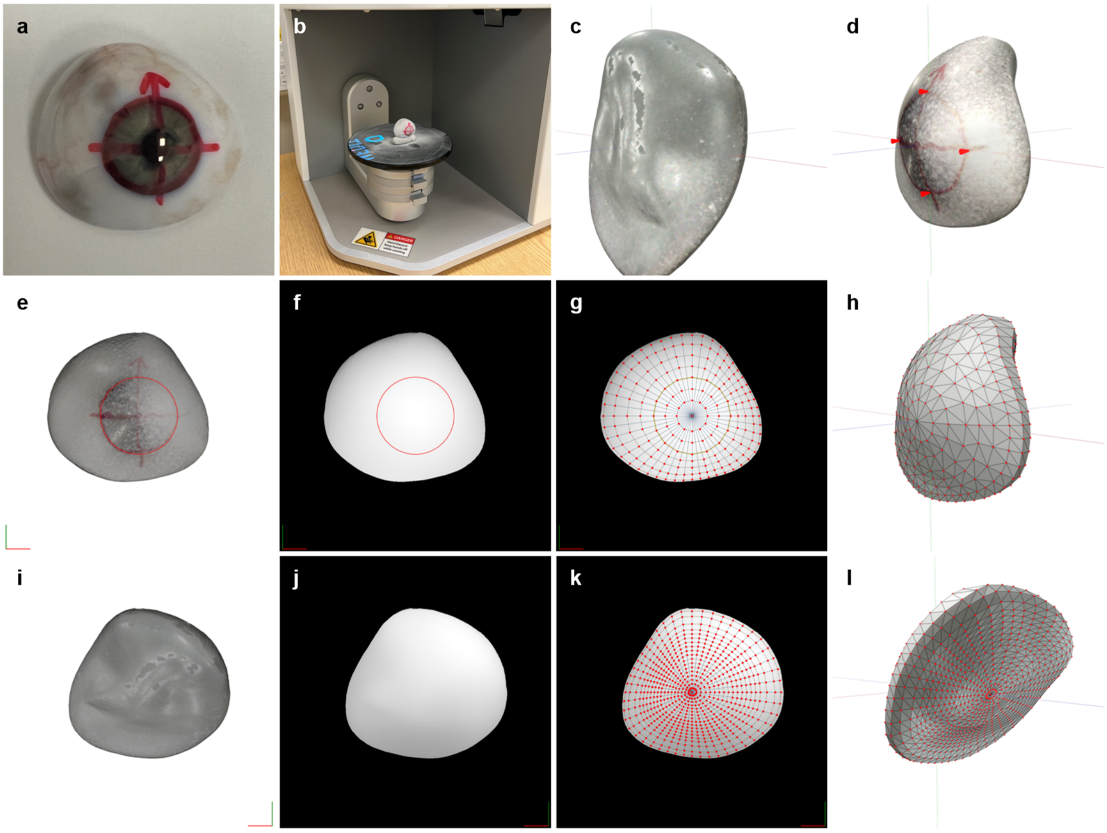

Supplementary Fig. 1 | Scan alignment and correspondence. a, Marked prosthesis, the arrow indicates the superior direction. **b,** The marked prosthesis in the Medit T500 dental 3D-scanner. **c,** A 3D Scan of a prothesis. **d,** The mesh after alignment, the intersection points of the ring and crosshair (shown in red) lie on the x-axis or y-axis respectively. **e,f,** orthographic projection (**e**) and depth map (**f**) of the anterior surface or front of the prosthesis shape. **i,j,** orthographic projection (**i**) and depth map (**j**) of the posterior surface or back of the prosthesis. **g,k,** Correspondences for front (**g**) and back (**k**) of the prosthesis, the feature points (red dots) are determined with line tracing on the depth map; blue lines indicates the radial lines for the line tracing and the yellow loops connect points that are placed at the same ratio on these lines. Note that points are placed only on every second or third segment for smaller ratios to keep the density homogeneous, points on the back are placed with a higher density since the shape variation on the back has a higher frequency. Also note that for the front we ensure that the third loop lies on the limbus. **h,l,** resulting shape of the prosthesis with the vertices marked in red.

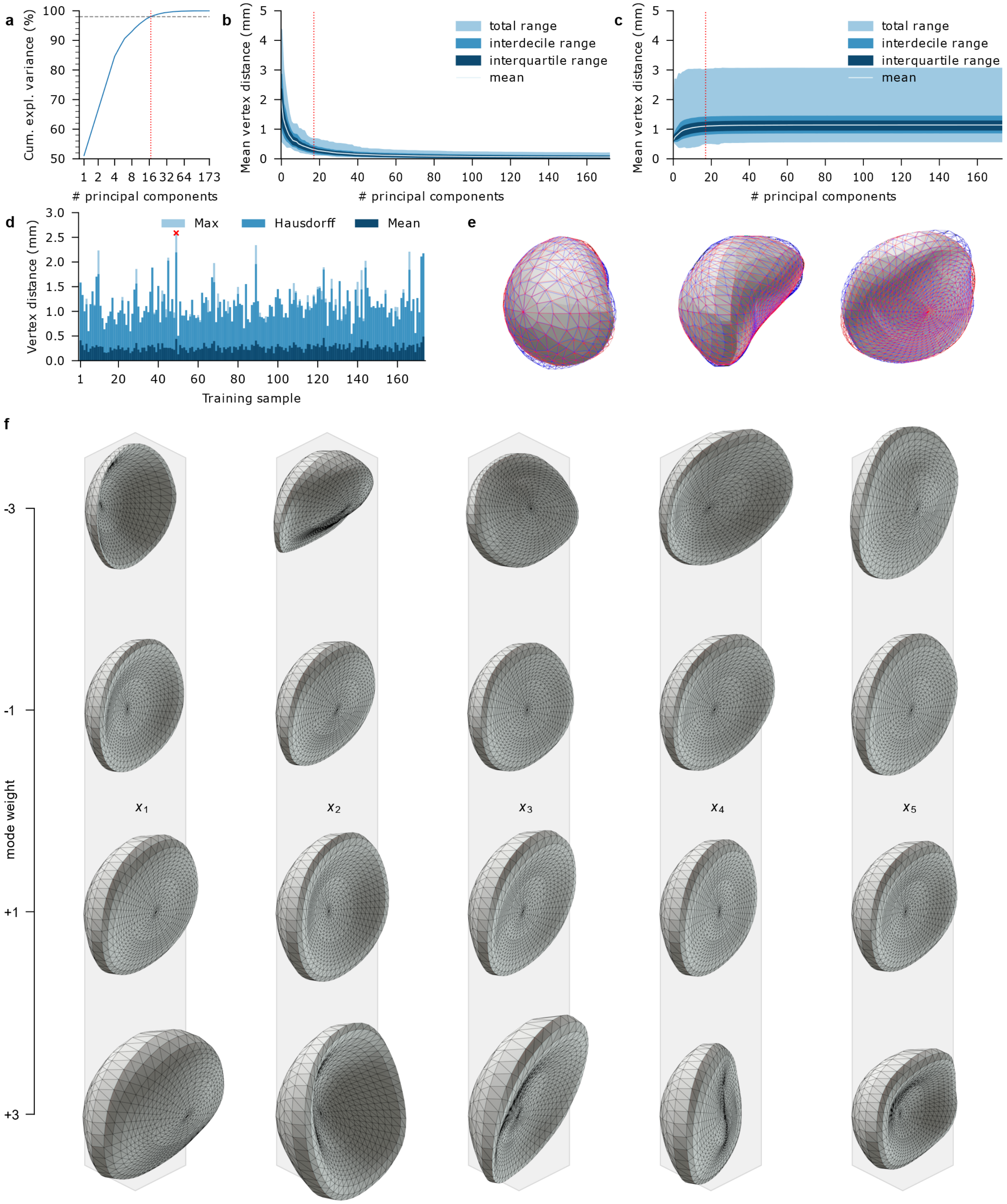

Supplementary Fig. 2 | Statistical shape model. a, Shape model compactness represented by the cumulative explained shape variance of the principal components or modes, already the first 17 of 173 modes (red dotted line) explain about 98% of the variance (grey dashed line); note the inverse hyperbolic scale of the x-axis. **b,** Generalization, given by the mean vertex distance error to one shape excluded from the training set, decreases as expected with the number of modes. **c,** Specificity, given by the smallest mean vertex distance error of a set of 10.000 randomly sampled shapes to any shape of the training set, remains largely constant. **d,** Reconstruction errors, measured as the vertex distance of corresponding vertices, of the training data for the shape model using the first 17 modes; the sample with the highest maximal error of 2.59 mm and Hausdorff distance of 2.20 mm is marked in red. **e,** Comparison of original geometry (blue wireframe) of the sample with the highest reconstruction error to the geometry of its representation in the shape model (red wireframe) from different perspectives, with the intersection as white solid. **f,** Impact on shape and size of the first five principal components or modes (left to right) visualized for four weights (top to bottom), each shape is $S(w\,\mathbf{x}_j)$ with $\mathbf{x}_j = (\delta_{j\,1}, \delta_{j\,2}, \ldots, \delta_{j\,17})^T$, the components of Kronecker deltas (being zero for all components except the mode j), with weight $w \in \{-3, -1, 1, 3\}$. These first five modes cover already about 90% of the shape variations.

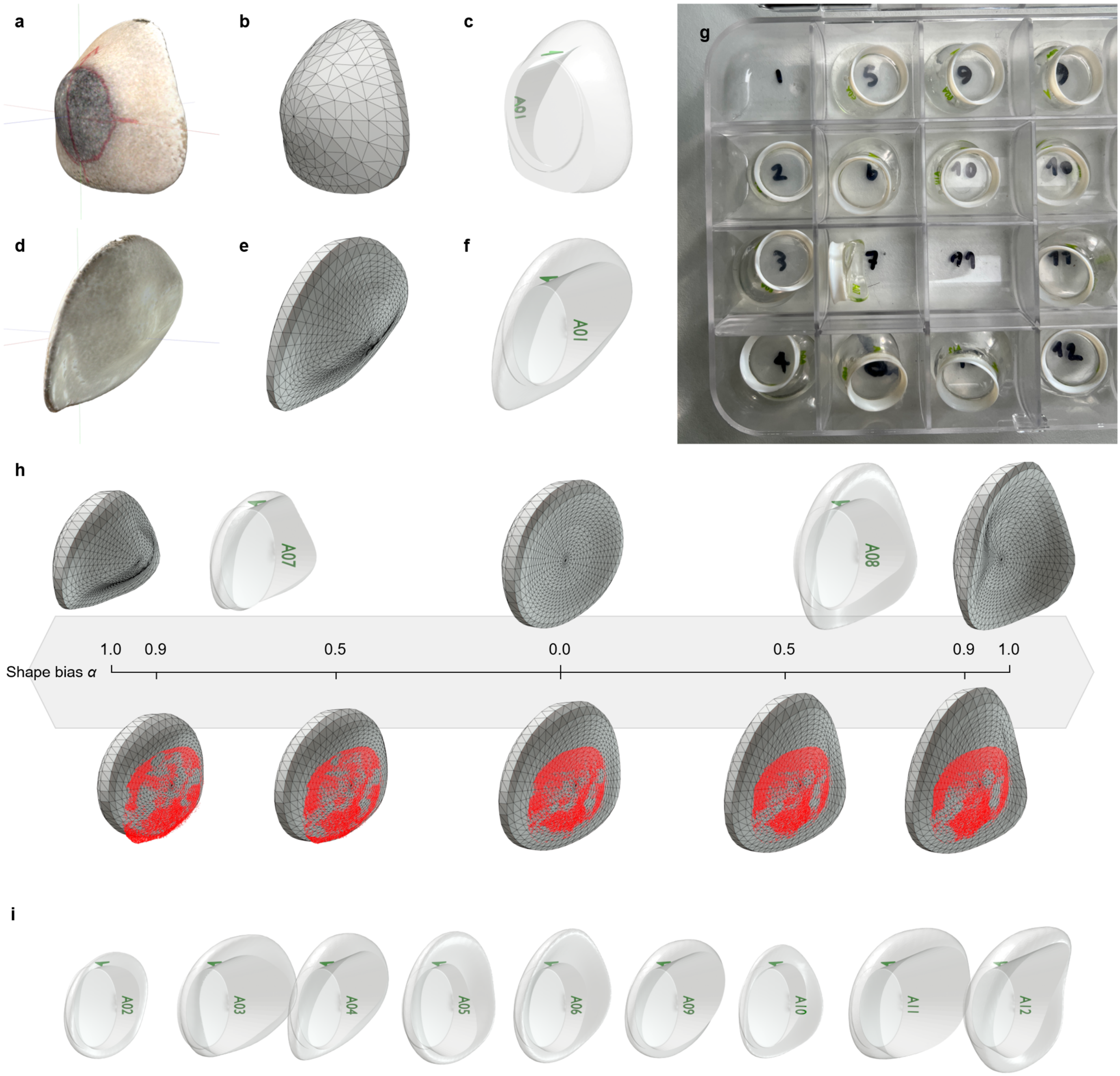

Supplementary Fig. 3 | Conformer design and influence on shape. a,d, Original shape of the prosthesis (after alignment) used for conformer A01, from front (**a**) and back (**d**). **b,e,** Base shape $S(\mathbf{x}_1)$ derived from its representation in the SSM, from front (**b**) and back (**e**). **c,f,** Conformer A01 design with planar surface in the front and cut out frustum on the back. **g,** Conformers printed with a J750 3D printer, note the additional white lid lift to hold the eyelids open; each of the 12 conformers was printed both for left and right eyes. **h**, Impact of the conformer choice and bias α on the predicted shape $S(\mathbf{x}_f)$ for the same socket surface of Patient 1 (lower half), mean shape bias $\alpha = 0.0$ in the centre, shapes biased towards conformer A07 (shown left with base shape $S(\mathbf{x}_7)$) to the left, and towards conformer A08 to the right (shown right with $S(\mathbf{x}_8)$). Each predicted shape fits the socket surface well, the conformer bias mostly affects the size and edges of the prosthesis shape; note that the energy weights the surface distance to the socket higher than the similarity to the target shape. **i**, Conformers A02 to A06 and A09 to A12 (left to right).

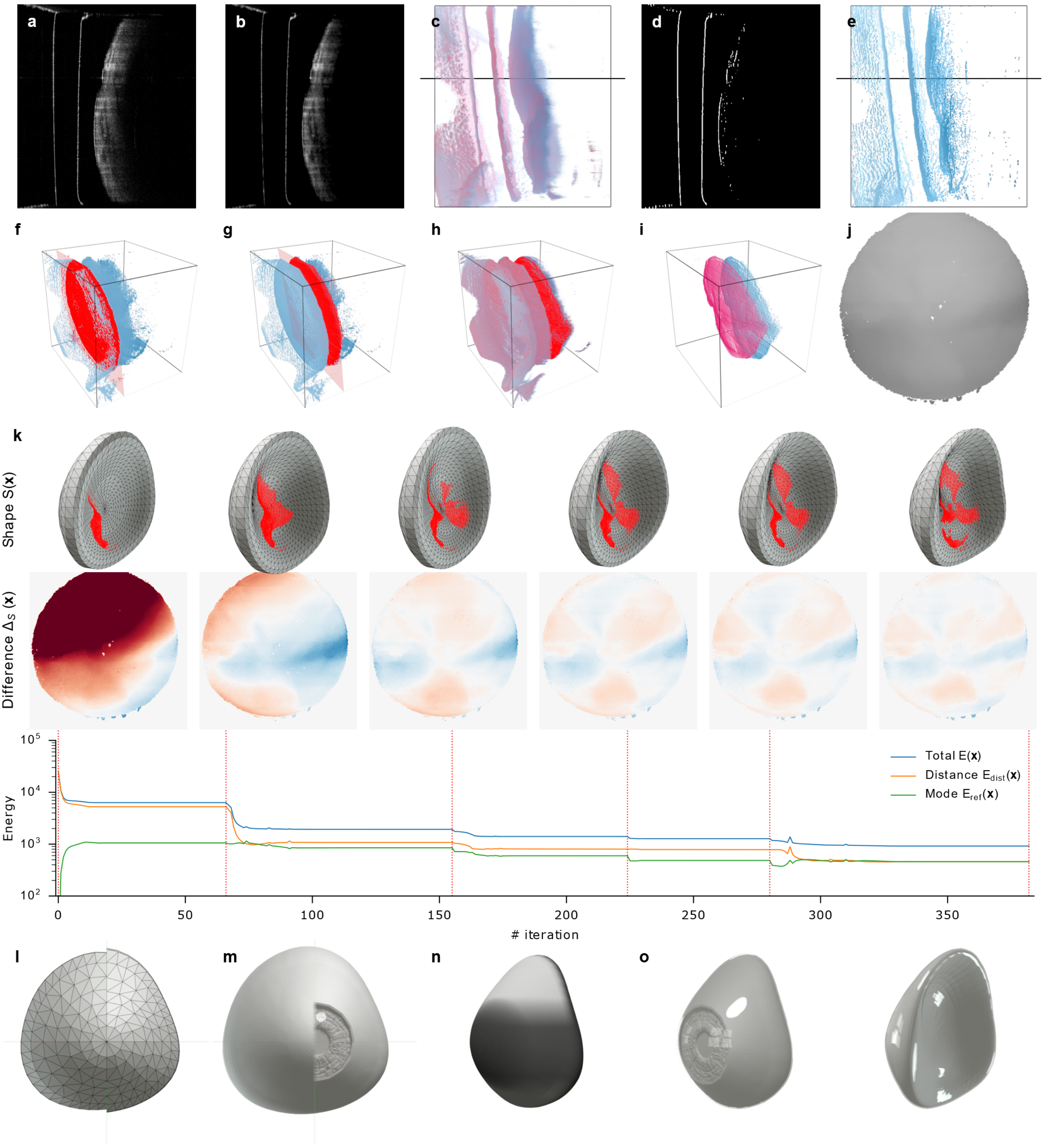

Supplementary Fig. 4 | Socket surface extraction from the OCT data and shape prediction for the prosthesis. a, One of the 256 OCT slices $\mathbf{B}_{\text{Input}}$ of Patient 4, with top-down corresponding to the temporal-nasal axis and left-right to the depth. **b,** Median filtered and downscaled $\mathbf{B}_{\text{Median}}$, note disappearing segments in the window surface. **c,** Orthographic rendering of the volumetric data V_{Median}, the position of the slice (**b**) is marked with a black line. **d,e,** Max Pooling result $\mathbf{B}_{\text{Max}}$ with the window surface clearly visible(**d**), merged into volumetric data set V_{Edge} (**e**). **f-i,** Extraction of the anophthalmic socket surface. Column tracing of V_{Edge} yields the points of the anterior surface of the window and fitted plane (**f**, red) and points of the posterior surface of the window with the fitted plane (**g**, red). Column tracing of V_{Median} yields socket surface points (**h**, red) which are corrected for the conformer geometry and angle of gaze (**i**, blue before, red after). **j,** Depth map $\mathbf{D}_{\text{S}}$ of the socket surface. **k,** Fitting of a shape $\text{S}(\mathbf{x})$ to the socket surface; starting with the target shape $\mathbf{x}_{\text{t}}$ the shape parameters $\mathbf{x}$ are varied to reduce the energy $\text{E}(\mathbf{x})$, plotted below with the different terms; the red lines indicate the iterations that enable additional modes for the shape parameter. The depth map difference $\Delta_{\text{S}}(\mathbf{x}) = \mathbf{D}_{\text{S}} - \text{Z}_{\text{z}}(\text{S}(\mathbf{x}))$ and the shape $\text{S}(\mathbf{x})$ compared to the socket surface P_{S} is shown above for these iterations. The energy is steadily decreasing, leading to a better fit to the socket surface. **l,** Shape before (left) and after (right) post-processing, the shape is scaled at the edges, the limbus is resized to fit the iris, and the cornea is normalized to the mean shape. **m,** Shape after smoothing (left) and with the iris geometry (right). **n,** Clear coating thickness, brighter areas have thicker coating, up to 1 mm is added at the front. **o,** Final shape with simulated clear coating.

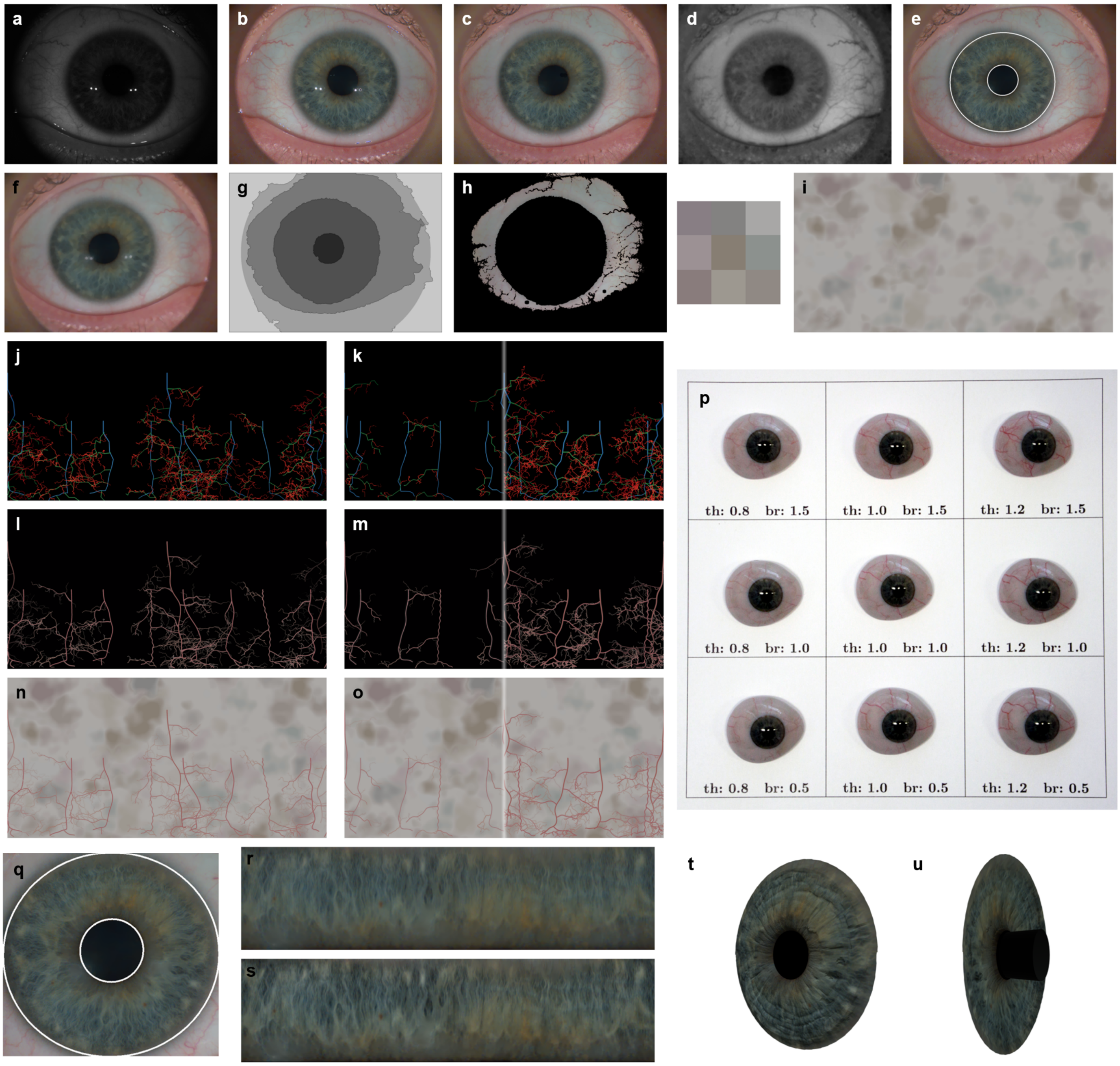

Supplementary Fig. 5 | Colour image processing and texture generation. **a**, Raw image I_{raw} of the companion eye from the OCT colour camera. **b**, Colour characterized and denoised image I_{cal}. **c**, Specular highlights removed in I_{clean}. **d**, The greyscale-enhanced image I_{enh} used to extract the iris region. **e**, Iris segmentation $\mathbf{M}_{\text{I}}$ using Daugman's algorithm. **f**, Median blurred image I_{median}. **g**, Watershed segmentation $\mathbf{M}_{\text{E}}$ of pupil, iris, sclera, and skin and camera aperture (dark to bright). **h**, Refined sclera mask $\mathbf{M}_{\text{W}}$ with the extract a set of sclera colours G. **i**, Sclera base texture with Perlin noise generated staining. **j,l,n**, A vein network (**j**) is grown procedurally in three layers (blue first, green second, and red third layer) using veining parameters, rendered using a set of vein profiles (**l**), and rendered on top of the sclera texture (**n**). **k,m,o,** Influence of the veining parameters on the generated texture, low veining parameters (left half) result in fewer and thinner veins as high parameters (right half) in the network (**k**), rendering (**m**), and texture (**o**). **p**, 3D prints of prosthesis models with different veining parameters for branching ratio *br* and thickness *th*; these prostheses were used by the ocularist as references to select the veining parameters. **q,r,s**, The iris region (**q**) is unwrapped in cylindrical coordinates (**r**) and contrast-enhanced in the lightness channel (**s**). **t**, The iris texture mapped on the iris geometry. **u**, A black cylinder is placed behind the pupil to increase the light absorption on the print.

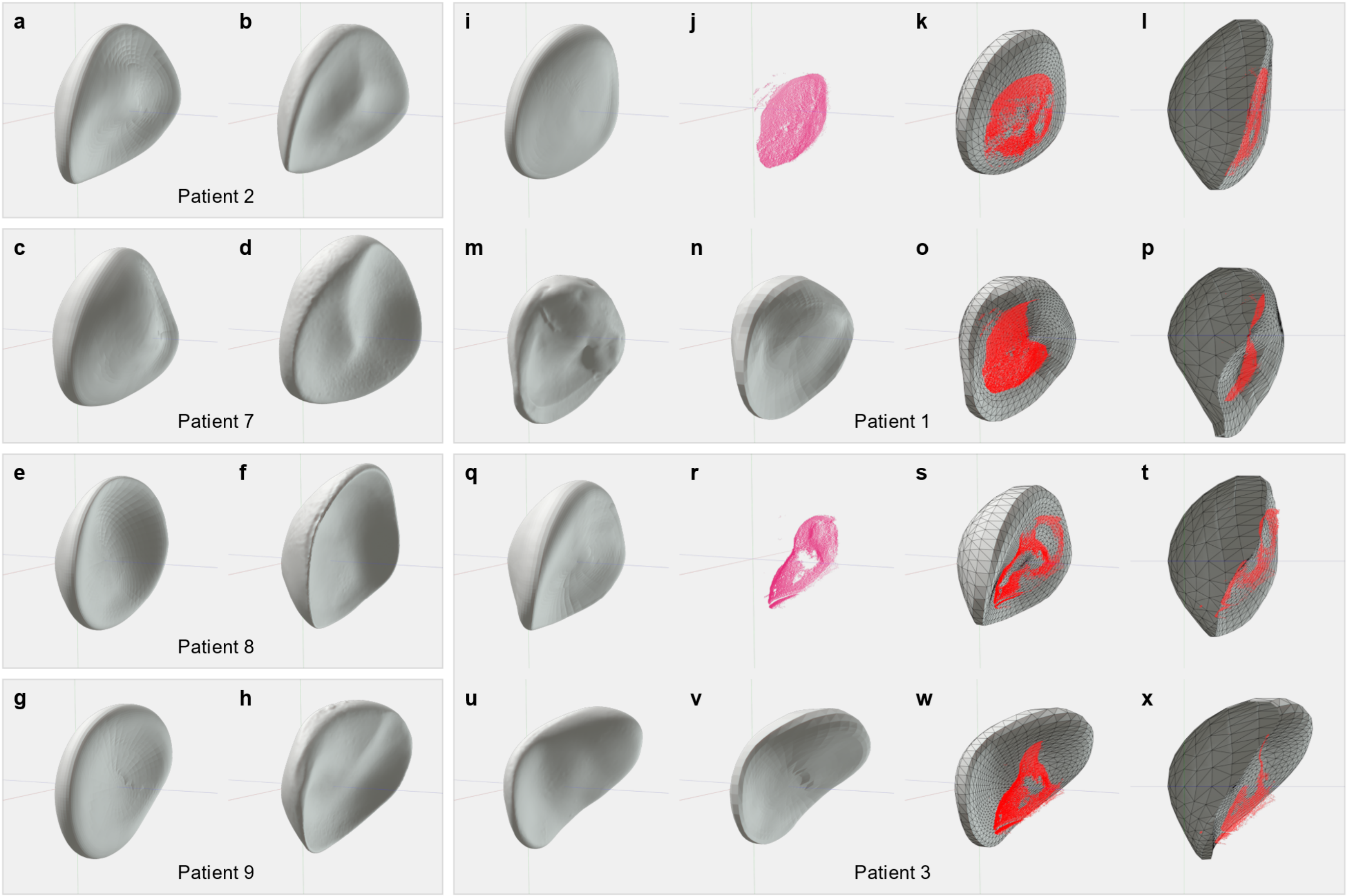

Supplementary Fig. 6 | Adjustments of the prostheses' shapes during the fitting. a,b, Prosthesis shape of Patient 2 before (**a**) and after (**b**) adjustment, the inferior edge was thinned. **c,d,** Prosthesis shape of Patient 7 before (**c**) and after (**d**) adjustment, material at the posterior surface was ground away. **e,f,** Prosthesis shape of Patient 8 before (**e**) and after (**f**) adjustment, the edges have been thinned. **g,h,** Prosthesis shape of Patient 9 before (**g**) and after (**h**) adjustment, the inferior edge has been thinned. **i-p,** Comparing the predicted shape $S(\mathbf{x}_f)$ for Patient 1 (**i**) to the supplied manually modified and wax-adjusted shape(**m**) shows that, even though it fits the extracted surface (**j**) very well (**k,l**) and the shape model can reasonably replicate the unusual shape of the hand-made prosthesis (**n**), the predicted shape is very different, because the extracted surface does not match the posterior surface of the hand-made shape (**o**, orthographic cross-section **p**). **q-x**, Comparing the predicted shape $S(\mathbf{x}_f)$ for Patient 3 (**q**) and the hand-made shape (**u**) shows the same observation. Note that for Patients 4, 5, 6, and 10 only minor adjustments were made.

List of symbols

- α : Shape bias
- $\mathbf{B}_{\mathrm{Input}}$: OCT bitmap slice image
- $\mathbf{B}_{\mathrm{Median}}$: Filtered OCT image
- $\mathbf{B}_{\mathrm{Edge}}$: Binary edge map
- c : Conformer
- $\mathbf{D}_{\mathrm{A}}$: Depth map of anterior conformer surface
- $\mathbf{D}_{\mathrm{B}}$: Depth map of posterior conformer surface
- $\mathbf{D}_{\mathrm{P}}$: Depth map of anterior surface plane fit
- $\mathbf{D}_{\mathrm{S}}$: Depth map of socket surface
- $\mathrm{E}(\mathbf{x})$: Energy
- $\mathcal{F}$: Faces of the shape
- G : Set of sclera colours
- I_{clean} : Clean colour image
- I_{col} : Colour characterized image
- I_{enh} : Greyscale enhanced contrast image
- I_{median} : Median blurred colour image
- I_{raw} : Raw colour camera image
- I_{S} : Masked sclera image
- k : Number of modes
- m : Scanned mesh
- $\mathbf{M}_{\mathrm{A}}$: Binary mask of anterior conformer surface
- $\mathbf{M}_{\mathrm{B}}$: Binary mask of posterior conformer surface
- $\mathbf{M}_{\mathrm{E}}$: Watershed segmentation of the colour image
- $\mathbf{M}_{\mathrm{H}}$: Binary mask of specular highlights
- $\mathbf{M}_{\mathrm{I}}$: Daugman iris segmentation
- $\mathbf{M}_{\mathrm{S}}$: Mask of socket surface
- $\mathbf{M}_{\mathrm{W}}$: Refined segmentation of the sclera
- $\mathrm{N}_{\mathrm{P}}(x, y, z)\colon \mathbb{R}^3 \mapsto \mathbb{R}$: 3D Perlin noise
- P_{A} : Plane of the anterior conformer surface
- P_{S} : Point cloud of socket surface
- $\mathcal{P}$: Point cloud of the shape, i.e. shape without faces
- $\mathrm{S}(\mathbf{x})\colon \mathbb{X} \mapsto \Theta$: Shape model
- $\mathcal{S}$: Shape with faces
- $\mathcal{S}_{\mathrm{m}}$: Mean shape
- $\mathcal{S}_c$: Scanned conformer shapes
- $\mathcal{S}_{\mathrm{rec}}$: Rec shape, based on a 3D scan
- τ : Conformer thickness (in pixels)
- Θ : Space of meshes with faces
- T_I : Unwrapped iris texture
- V_{Median} : Volumetric data
- V_{Edge} : Volumetric edge data
- w_{dist} : Weight of distance term
- $\mathbf{w}_{\mathrm{ref}}$: Weights of reference term
- $\mathbb{X} = \mathbb{R}^k$: Shape space
- x_i : i^{th} mode
- $\mathbf{x}_c$: Shape vector of conformer c (Base shape)
- $\mathbf{x}_{\mathrm{f}}$: Solution of fitting
- $\mathbf{x}_{\mathrm{m}}$: Mean shape
- $\mathbf{x}_{\mathrm{t}}$: Target shape
- $\mathrm{Z}_{\mathrm{z}}(\mathcal{S})\colon \Theta \mapsto \mathbb{R}^{256\times256}$: Orthographic depth-map z-projection

Algorithm pseudocode

Algorithm 1: Alignment of scan data

<u>function align-mesh</u> $(V_I, F_I, s, p_S, p_N, p_I, p_T)$:
Input: 3D mesh with vertices V_I and faces F_I, eye side s (‘L’ or ‘R’), and four pick point positions p_S, p_N, p_I, p_T
Output: Aligned 3D mesh with vertices V_A and faces F_A and estimated iris radius r
$r_S, r_N, r_I, r_T = (1,0,0)^T, (0,1,0)^T, (-1,0,0)^T, (0,-1,0)^T$;
# Find homogeneous transformation matrix that maps pick points to reference points
$c = (p_S + p_N + p_I + p_T) / 4$;
$p_S, p_N, p_I, p_T = p_S - c, p_N - c, p_I - c, p_T - c$;
# Compute rotation matrix using least squares
$R = \operatorname{argmin}(\| [p_S, p_N, p_I, p_T]^T R - [r_S, r_N, r_I, r_T]^T \|_2)$;
$T = \begin{bmatrix} R & Rc \\ (0,0,0) & 1 \end{bmatrix}$
# Mirror along z-axis if left eye, since rotation yields flipped orientation
if s == ‘L’:
    $T[3,3] = -T[3,3]$;
$V_A = \emptyset$;
for v **in** V_I:
    $V_A = V_A \cup (T\, v)$;
# Flip faces if left eye
$F_A = F_I$;
if s == ‘L’:
    for f **in** F_A:
        $f = (f[2], f[1], f[0])$;
$r = (\|T\, p_S\|_2 + \|T\, p_N\|_2 + \|T\, p_I\|_2 + \|T\, p_T\|_2) / 4$;
return V_A, F_A, r

Algorithm 2: Computing correspondences

<u>function render-depth-map</u> $(V, F, \vec{d})$:
Input: 3D mesh with vertices V and faces F and direction $\vec{d}$
Output: Depth map from orthogonal projection along direction $\vec{d}$

<u>function correspondences</u> (V_A, F_A, r):
Input: Aligned 3D mesh with vertices V_A and faces F_A and estimated iris radius r
Output: 3D point cloud with sequence S of 838 corresponding vertices
# Render orthographic depth maps of front and back of prosthesis mesh
D_F = render-depth-map($V_A, F_A, (0,0,-1)$);
D_B = render-depth-map($V_A, F_A, (0,0,1)$);
# Trace depth map from origin via limbus to edges and store limbus and edge position
$s_{\text{seg}} = 48$;
$V = ()$;
for i **in** $\{0, \ldots, s-1\}$:
  $\theta = 2\pi\ i/s_{\text{seg}}$;
  $d_x, d_y = (-\sin\theta, \cos\theta)$;
  $l_x, l_y = (r\ d_x, r\ d_y)$;
  $e_x, e_y = (l_x,\ l_y)$;
  while $D_F[e_x + d_x, e_y + d_y] \neq \infty$:
    $e_x, e_y = (e_x + d_x,\ e_y + d_y)$;
  $V = V^\frown((l_x, l_y, e_x, e_y))$;
# Add cornea points by iterating over segments and rings up to limbus
$S = ((0, 0, D_F[0,0])^T)$;
for q_c, s_c **in** $((0.4, 4), (0.7, 3), (1.0, 3))$:
  for i **in** $\{x \in s_c\mathbb{N}_0 : x < s_{\text{seg}}\}$:
    $l_x, l_y, e_x, e_y = V[i]$;
    $p_x, p_y = (l_x\ q_c, l_y\ q_c)$;
    $S = S^\frown((p_x, p_y, D_F[p_x, p_y])^T)$;
# Add remaining points of the front
for q_c, s_c **in** $((0.25, 2), (0.5, 2), (0.75, 1), (0.95, 1))$:
  for i **in** $\{x \in s_c\mathbb{N}_0 : x < s_{\text{seg}}\}$:
    $l_x, l_y, e_x, e_y = V[i]$;
    $p_x, p_y = (l_x + q_c(e_x - l_x), l_y + q_c\ (e_y - l_y))$;
    $S = S^\frown((p_x, p_y, D_F[p_x, p_y])^T)$;
# Add points of the back of the prosthesis shape from edge to origin
for q_c, s_c **in** $((0.975, 1), (0.91, 1), (0.82, 1), (0.75, 1), (0.7, 1), (0.65, 1), (0.6, 1), (0.55, 1), (0.5, 1), (0.45, 1), (0.4, 2), (0.35, 2), (0.3, 2), (0.25, 2), (0.2, 2), (0.15, 3), (0.1, 3), (0.05, 3))$:
  for i **in** $\{x \in s_c\mathbb{N}_0 : x < s_{\text{seg}}\}$:
    $l_x, l_y, e_x, e_y = V[i]$;
    $p_x, p_y = (q_c\ e_x, q_c\ e_y)$;
    $S = S^\frown((p_x, p_y, D_B[p_x, p_y])^T)$;
$S = S^\frown((0, 0, D_B[0,0])^T)$;
return S;

Algorithm 3: Shape model creation

1. <u>function SSM</u> (Ψ):
   Input: Set M of 3D meshes, each with vertices V_I and faces F_I, eye side s ('L' or 'R'), and four pick point positions p_S, p_N, p_I, p_T
   Output: PCA-based statistical shape model $S(x)$
2. $X = \mathbb{R}^{0\times 3n}$;
3. **for** $V_I, F_I, s, p_S, p_N, p_I, p_T$ **in** M:
4. $\quad V_A, F_A, r = \mathrm{align}(V_I, F_I, s, p_S, p_N, p_I, p_N)$;
5. $\quad V_C = \mathrm{correspondences}(V_A, F_A, r)$;
6. $\quad X = (X|\mathrm{vec}(V_c))$;
   # Use principal component analysis to compute mean, components and eigenvalues
7. $\bar{x}, \Phi, \Lambda = \mathrm{PCA}(X)$;
   # Determine minimal number of modes to express 98% of the variance
8. $k = 1$;
9. **while** $\Sigma_{\mathrm{j=1}}^{\mathrm{k}} \lambda_j / \Sigma\lambda_i < 0.98$:
10. $\quad k = k + 1$;
    # Convert vectors into matrices of vertices
11. $\bar{x} = \mathrm{vec}_{\mathrm{n,3}}^{-1}(\bar{x})$;
12. **for** C_i **in** Φ:
13. $\quad \mathrm{C_i} = \mathrm{vec}_{\mathrm{n,3}}^{-1}(\mathrm{C_i})$;
14. **return** $S(\vec{x})\colon \mathbb{R}^k \mapsto \Theta = \bar{x} + \Sigma_{\mathrm{i=0}}^{\mathrm{k}}\, x_i \sqrt{\lambda_i}\, \mathrm{C}_i$;

Algorithm 4: Socket volume processing

1. function median-filter(I, k, r):
   Input: Gray-scale image I, kernel size k, repetitions r
   Output: Image after r-times application of median filter with kernel $k \times k$

2. function median-pool(I, k, r):
   Input: Image I, pool size k
   Output: Down-sampled image by factor k, each pixel result median filter with kernel $k \times k$

3. function max-pool(I, k, r):
   Input: Image I, pool size k
   Output: Down-sampled image by factor k, each pixel maximal value in kernel $k \times k$

4. function combine (B):
   Input: Set B of 256 gray-scale images of the raw OCT response
   Output: Volumetric data V_{Median} and V_{Edge} of size 536×256×469 voxels
5. $V_{\mathrm{Median}}, V_{\mathrm{Edge}} = \mathbb{R}^{535\times 0\times 469}, \mathbb{R}^{535\times 0\times 469}$;
6. **for** B_{Input} **in** B:
7. $B_T = (\max(B_{\mathrm{Input}}, 25) - 25)\ /\ (255 - 25)$;
8. $B'' = \text{median-filter}(B_T, 5, 2)$;
9. $B'' = \text{median-filter}(B'', 3, 2)$;
10. $B'' = \text{max-pool}(B'', 2)$;
11. $B'' = \text{median-filter}(B'', 3, 1)$;
12. $B'' = \text{median-pool}(B'', 2)$;
13. $B_1 = \text{median-filter}(B'', 3, 2)$;
14. $V_{\mathrm{Median}} = (V_{\mathrm{Median}} | B_1)$;
15. $B_2 = \text{max-pool}(B_T, 4)$;
16. $B_2 = B_2 \odot \mathbf{1}_{\{x \in B_1 : x > 0\}}(B_1))$;
17. $E = \partial^2 / \partial x^2\ B_2 < -0.05$;
18. $V_{\mathrm{Edge}} = (V_{\mathrm{Edge}} | E)$;
19. **return** $V_{\mathrm{Median}}, V_{\mathrm{Edge}}$;

Algorithm 5: Socket surface extraction

<u>function extract</u> ($V_{\text{Median}}, V_{\text{Edge}}, \tau$):

Input: Volumetric data V_{Median} and V_{Edge} of size 536×256×469 voxels, conformer window thickness τ in voxel.

Output: Socket surface depth map D_{S} and mask M_S of size 536×256 pixels, d_A distance of conformer anterior surface to image border in pixel

$D_A = \mathbb{R}^{535\times256}$;

# Trace depth map for front of conformer window

for x, y **in** $\{1, \dots, 535\} \times \{1, \dots, 256\}$:

  $z_1 = 1$;

  while $V_{\text{Edge}}[x, y, z_1] = 0$:

    $z_1 = z_1 + 1$;

  $D_A[x, y] = z_1$;

$t_A = \text{median}(D_A) + 0.75\tau$;

$M_A = D_A < t_A$;

# Refine mask by iterative fit of plane

$P = \mathbb{R}^{535\times256}$;

for i **in** $\{1, \dots, 8\}$:

  $a, b, c, d = \text{argmin}_{a,b,c,d} \Sigma_{\{\text{x,y: } \text{M}_\text{A}[x,y]=1\}} (D_A[x, y] - (ax + by - d)/(-c))^2$;

  for x, y **in** $\{1, \dots, 535\} \times \{1, \dots, 256\}$:

    $P[x, y] = (ax + by - d)/(-c)$;

  $M_A = |D_A - P| < 0.5\tau$;

# Depth of anterior conformer window

$d_A = P[267, 128]$;

# Median filter and compute closing of mask to remove gaps

$M_A = \text{median-filter}(M_A, 5, 1)$;

$E = \{(x, y) \in \mathbb{Z}^2 : x^2 + y^2 \leq 5^2\}$;

$M_A = (M_A \oplus E) \ominus E$;

# Trace depth map for back of conformer window

$D_B = D_A + 0.5\tau$;

for x, y **in** $\{1, \dots, 535\} \times \{1, \dots, 256\}$:

  $z_1 = D_B[x, y]$;

  while $V_{\text{Edge}}[x, y, z_1] = 0$:

    $z_1 = z_1 + 1$;

  $D_B[x, y] = z_1$;

$M_B = M_A$;

# Refine mask by iterative fit of plane

for i **in** $\{1, \dots, 4\}$:

  $a, b, c, d = \text{argmin}_{a,b,c,d} \Sigma_{\{\text{x,y: } \text{M}_\text{B}[x,y]=1\}} (D_B[x, y] - (ax + by - d)/(-c))^2$;

  for x, y **in** $\{1, \dots, 535\} \times \{1, \dots, 256\}$:

    $P[x, y] = (ax + by - d)/(-c)$;

  $M_B = |D_B - P| < 0.5\tau$;

# Median filter and compute closing of mask to remove gaps

$M_B = \text{median-filter}(M_B, 5, 1)$;

$E = \{(x, y) \in \mathbb{Z}^2 : x^2 + y^2 \leq 5^2\}$;

$M_B = (M_A \oplus E) \ominus E$;

$M_B = \text{median\_filter}(M_B, 5, 2)$;

$M_B = M_A \,\&\, M_B$;

# Trace depth map for socket surface

$D_S = D_B + 10$;

for x, y **in** $\{1, \dots, 535\} \times \{1, \dots, 256\}$:

```
    if M_B[x, y] = 0:
        continue;
    z_0 = D_S[x, y];
    z_1 = z_0;
    for z in {z_1 + 1, … , 469}:
        if V_Median[x, y, z] > V_Median[x, y, z_1]:
            z_1 = z;
    v_0 = V_Median[x, y, z_0];
    v_1 = V_Median[x, y, z_1];
    if v_1 > v_0:
        for z in {z_1 − 1, … , z_0 + 1}:
            v = (v_1 − v_0) / (z_1 − z_0) (z − z_0);
            if V_Median[x, y, z] > v:
                z_1 = z;
                v_1 = v;
    for z in {z_1, … , z_0}:
        if V_Median[x, y, z] = 0:
            D_S[x, y] = z;
            break;
  # If the median filtered depth differs too much it's probably an outlier
D*_S = median-filter(D_S, 5, 1);
for x, y in {1, … , 535} × {1, … ,256}:
    if |D*_S[x, y] − D_S[x, y]| > 16:
        D_S[x, y] = 0;
M_S = D_S > 0;
M_S = median-filter(M_S, 5, 1)& M_S;
return D_S, M_S, d_A;
```

Algorithm 6: Socket surface post-processing

1. function correct-conformer ($\vec{n}, t, s$):
   Input: surface normal $\vec{n}$ of the conformer in physical space, t conformer window thickness in mm, s conformer window offset in mm
   Output: Correction vector c
2. $n_1, n_2 = 1.00027, 1.5$;
3. $\vec{o} = (0,0,-1)^T$;
4. $\vec{p} = \vec{n} \times \vec{o}$;
5. $\theta_1 = \cos^{-1}(\vec{o} \cdot \vec{n})$;
6. $\theta_2 = \sin^{-1}(\sin(\theta_1)\, n_1/n_2)$;
   # Linear equation system to find refracted vector r
   *# Angle between n and r should be refracted angle : acos(n * r) = theta_2*
   *# Angle between o and r should be difference of angles : acos(o * r) = theta_1 – theta_2*
   *# Refracted r should be orthogonal on (n x o) * r = 0*
7. $A = \begin{pmatrix} n[1] & n[2] & n[3] \\ o[1] & o[2] & o[3] \\ p[1] & t[2] & p[3] \end{pmatrix}$;
8. $\vec{b} = (\cos(\theta_2), \cos(\theta_1, \theta_2), 0)^T$;
9. $\vec{r} = A^{-1}\vec{b}$;
10. $\vec{r} = \vec{r}/\|\vec{r}\|_2$;
11. $t = t\cos(\theta_1)\,/\,n_2$;
    # Correction for optical path change due to conformer tilt
12. $\vec{c}_R = (\vec{r} - \vec{o})\, t$;
    # Correction for conformer offset and empirical correction of OCT signal response
13. $\vec{c}_C = (s\,\vec{n}) + (0, 0, -0.35\, t)^{\mathrm{T}}$;
14. **return** $\vec{c}_R + \vec{c}_C$;

15. function correct ($D_S, M_S, \vec{n}, t, s, d_A$):
    Input: Socket surface depth map D_S and mask M_S of size 536×256 pixels, surface normal $\vec{n}$ of the conformer in physical space, t conformer window thickness in mm, s conformer window offset in mm, , d_A distance of conformer anterior surface to image border in mm
    Output: Corrected depth map D_S and mask M_S
    # Correct and compensate conformer impact
16. $\vec{c} = \text{correct-conformer}(\vec{n}, t, s)$;
    # Shift such that conformer anterior center at origin
17. $\vec{c} = \vec{c} + (0, 0, -d_A)^T$;
    # Correct angle of gaze with rotation around y-axis using estimated value
18. $R = R_y(6° \cdot \pi/180)$;
    # Convert depth map to point cloud and apply correction
19. $P = \emptyset$;
20. **for** x, y **in** $\{1, \dots, 535\} \times \{1, \dots, 256\}$:
21.   **if** $M_S[x, y] > 0$:
22. $P = P \cup \{R\,((x, y, D_S[x, y])^T + \vec{c})\}$;
    # Convert point cloud back to depth map
23. D_S = render-depth-map(P, (0,0,1));
24. $M_S = D_S \neq \infty$;
25. **return** D_S, M_S;

Algorithm 7: Shape fitting

<u>function energy-dist</u>(D_x, D_S, M_S):
Input: Shape depth map D_x, socket surface depth map D_S and mask M_S
Output: Scalar energy E_d
    # Compute sum of pixel-wise squared differences, normalize by mask area
$E_d = 0$;
for x, y **in** $\{1, \dots, 256\} \times \{1, \dots, 256\}$:
    if $M_S[x, y] > 0$:
        $E_d = E_d + (D_x[x, y] - D_S[x, y])^2$;
$E_d = E_d \cdot 10000 \,/\, \Sigma_{i \in M_S} i$;
return E_d;

<u>function energy</u>($\vec{x}, D_S, M_S, \vec{x}_t$):
Input: Shape vector $\vec{x}$, corrected depth map D_S and mask M_S, target shape $\vec{x}_t$
Output: Scalar energy $E(\vec{x})$
# Render orthographic depth map of SSM shape's back using the common faces
$D_x = \text{render-depth-map}(S(\vec{x}), \mathcal{F}, (0,0,1))$;
$E_d = \text{energy-dist}(D_x, D_S, M_S)$;
$\vec{w}_{\text{ref}} = (2048, 1024, 512, 256, 128, 64, 32, 16, 16, 16, \dots)^{\text{T}}$;
$E_r = \|\vec{w}_{\text{ref}}(\vec{x} - \vec{x}_t)\|_2$;
return $E_d + E_r$;

<u>function energy</u>($\vec{x}, \theta, z, D_S, M_S, \vec{x}_t$):
Input: Shape vector $\vec{x}$, rotation θ, translation in z-axis z, corrected depth map D_S and mask M_S, target shape $\vec{x}_t$
Output: Scalar energy $E(\vec{x})$
# Render orthographic depth map of SSM shape's back using the common faces
$D_x = \text{render-depth-map}(R_y(\theta)\, S(\vec{x}) + (0,0,z)^T, \mathcal{F}, (0,0,1))$;
$E_d = \text{energy-dist}(D_x, D_S, M_S)$;
$\vec{w}_{\text{ref}} = (2048, 1024, 512, 256, 128, 64, 32, 16, 16, 16, \dots)^{\text{T}}$;
$E_r = \|\vec{w}_{\text{ref}}(\vec{x} - \vec{x}_t)\|_2$;
return $E_d + E_r + 10000\,(\theta^2 + z^2)$;

<u>function fit-shapes</u> ($D_S, M_S, \vec{x}_c$):
Input: Corrected depth map D_S and mask M_S, conformer shape $\vec{x}_c$
Output: Set of fitted shapes $\{S(x)\}$
$X = \emptyset$;
for α **in** $\{0.0, 0.5, 0.9\}$:
    $\vec{x}_t = \alpha \vec{x}_c$;
    $\vec{x}_f = \vec{x}_t$;
    # Iteratively minimize energy using L-BFGS, using the first m modes
    for m **in** $\{3, 6, 9, 13, 17\}$:
        $\vec{x}_f = \text{L-BFGS}(\text{energy}(\vec{x}_f[1{:}m], D_S, M_S, \vec{x}_t[1{:}m]))$;
        # Change target shape bias towards current shape
        for i **in** $\{1, m\}$:
            $x_t[i] = x_t[i] - 0.1\,(x_t[i] - x_f[i])$;
    $X = X \cup \{\vec{x}_f\}$;
# Check if results are unlikely and allow translation and rotation during fit
if $\exists \vec{x} \in X : \text{absmax}\, \vec{x} \geq 3$:
    $X = \emptyset$;
    for α **in** $\{0.0, 0.5, 0.9\}$:
        $\vec{x}_t = \alpha \vec{x}_c$;

$\vec{x}_f, \theta, z = \vec{x}_t, 0, 0;$

# Iteratively minimize energy with transform using L-BFGS, using the first m modes

for m **in** $\{3, 6, 9, 13, 17\}$:

$\vec{x}_f = \text{L-BFGS}\big(\text{energy}\big(\vec{x}_f[1{:}m], \theta, z, D_S, M_S, \vec{x}_t[1{:}m]\big)\big);$

# Change target shape bias towards current shape

for i **in** $\{1, m\}$:

$x_t[i] = x_t[i] - 0.1\,(x_t[i] - x_f[i]);$

$X = X \cup \{\vec{x}_f\};$

# Convert to 3D shapes

return $= \{S(\vec{x}) : \vec{x} \in X\};$

Algorithm 8: Shape post-processing

<u>function post_process</u> (V_S, V_M, r_I, ρ):
Input: Shape vertice V_S, mean shape mesh V_M, iris radius r_I, boolean ρ indicating if reconstructed shape
Output: PCA-based statistical shape model $S(x)$
$s_{\text{seg}} = 48;$
$s_c = (4,3,3,2,2,1,1);$
$s_B = (1,1,1,1,1,1,1,1,1,1,2,2,2,2,2,3,3,3);$
$i_c = 1 + \Sigma_{i\in\{1,2,3\}}\ s_{\text{seg}}/(s_c[i]);$
# Set cornea to mean shape and smooth transition
for i **in** $\{1,\dots,i_c\}$:
  $V_S[i] = V_m[i];$
$r_l, r_n, r_B = 4, 7, 18;$
for r **in** $\{\mathrm{r_l},\dots,\mathrm{r_n}\}$:
  $s = ((r_n - r - 1)/(r_n - r_l))^2;$
  for _ **in** $\{1,\dots,s_{\text{seg}}/(s_c[r])\}$:
    $V_S[i] = (1-s)\ V_S[i] + s\ V_M[i];$
    $i = i + 1;$
# Inflate shape if not reconstructed shape
if ρ:
  for r **in** $\{\mathrm{r_l},\dots,\mathrm{r_n}\}$:
    $s = (r - r_l)/(r_n - r_l);$
    for _ **in** $\{1,\dots,s_{\text{seg}}/(s_c[r])\}$:
      $V_S[i] = V_S[i] \odot (1+0.05s, 1+0.05s, 1+0.05s)^T;$
      $i = i + 1;$
  for r **in** $\{1,\dots,r_B\}$:
    for _ **in** $\{1,\dots,s_{\text{seg}}/(s_B[r])\}$:
      $V_S[i] = V_S[i] \odot (1.05, 1.05, 1.05)^T;$
      $i = i + 1;$
# Scale cornea to fit iris radius and have cornea apex of 2.5 mm
$n_l = s_{\text{seg}}/(s_c[3]);$
$d_I = \Sigma_{i\in\{i_c - n_l,\dots,i_c\}} \|V_S[i]\|_2\ /\ n_l;$
$d_C = \|V_S[0]\|_2;$
for i **in** $\{1,\dots,i_c\}$:
  $V_S[i] = V_S[i] \odot (r_I/d_I, r_I/d_I, 2.5/d_C)^T;$
for r **in** $\{\mathrm{r_l},\dots,\mathrm{r_n}\}$:
  $s = (r_n - r - 1)/(r_n - r_l);$
  for _ **in** $\{1,\dots,s_{\text{seg}}/(s_c[r])\}$:
    $V_S[i] = V_S[i] \odot (r_I/d_I, r_I/d_I, 1)^T;$
    $i = i + 1;$
return V_S;

Algorithm 9: Compute color characterization

<u>function preprocess_color_image</u> $(I_{\mathrm{RGB}}, B_{\mathrm{raw}}, W_{\mathrm{raw}})$:
Input: Color image I_{RGB} to be pre-processed, raw color image B_{raw} taken without light (camera cover closed), raw color image W_{raw} taken of a uniform white patch
Output: Processed color image I_{RGB}

$w, h = 1280, 1024;$
# Demosaic images
$B = \mathrm{demosaic}(B_{\mathrm{raw}}, \text{'GRBG'});$
$W = \mathrm{demosaic}(W_{\mathrm{raw}}, \text{'GRBG'});$*# Black noise removal, compute mean value and subtract*
$\bar{b} = \Sigma_{x,y \in \{w/4,\ldots,w/4+w/2\} \times \{h/4,\ldots,h/4+h/2\}} B[x,y] \cdot 4/(wh);$
for x, y **in** $\{1, \ldots, w\} \times \{1, \ldots, h\}$:
  $I_{\mathrm{RGB}}[x,y] = I_{\mathrm{RGB}}[x,y] - \bar{b};$
  $W[x,y] = W[x,y] - \bar{b};$
# Flat fielding, compute mean value and rescale
$\bar{w} = \Sigma_{x,y \in \{w/4,\ldots,w/4+w/2\} \times \{h/4,\ldots,h/4+h/2\}} W[x,y] \cdot 4/(wh);$
for x, y **in** $\{1, \ldots, w\} \times \{1, \ldots, h\}$:
  $I_{\mathrm{RGB}}[x,y] = \bar{w} \odot (I_{\mathrm{RGB}}[x,y] \oslash W[x,y]);$
return $I_{\mathrm{RGB}};$

<u>function compute_characterization</u> $(\mathcal{I}_C, R_{\mathrm{LAB}}, R_{\mathrm{xyz}})$:
Input: 63 linear RGB color images $\mathcal{I}_C$ of color patches after preprocessing, reference CIELAB R_{LAB} and CIEXYZ R_{xyz} colors
Output: Color characterization matrices A and B

# Compute average RGB value for each patch
$w, h = 1280, 1024;$
$X_{\mathrm{RGB}} = \mathbb{R}^{0\times 3};$
for i **in** 63:
  $I_{\mathrm{RGB}} = \mathcal{I}_C[i];$
  $x = \Sigma_{x,y \in \{w/4,\ldots,w/4+w/2\} \times \{h/4,\ldots,h/4+h/2\}} I_{\mathrm{RGB}}[x,y] \cdot 4/(wh);$
  $X_{\mathrm{RGB}} = (X_{\mathrm{RGB}} | x);$
# Compute least squares solution of matrix A
$A = \mathrm{argmin}(\| X_{\mathrm{RGB}}\, A - R_{\mathrm{XYZ}} \|_2);$
# Convert to CIEXYZ then to CIELAB and store polynomial
$X_{\mathrm{LAB}} = \mathbb{R}^{0\times 7};$
for i **in** 63:
  $x_{\mathrm{RGB}} = X[i];$
  $x_{\mathrm{XYZ}} = x_{\mathrm{RGB}}\, A;$
  $L^*, a^*, b^* = \mathrm{CIELAB}(x_{\mathrm{XYZ}}, \mathrm{D50}, 2°);$
  $x = (L^*, a^*, b^*, \sqrt{L^* a^*}, \sqrt{L^* b^*}, \sqrt{a^* b^*}, 1);$
  $X_{\mathrm{LAB}} = (X_{\mathrm{LAB}} | x);$
# Compute least squares solution of matrix B
$B = \mathrm{argmin}(\| X_{\mathrm{LAB}}\, B - R_{\mathrm{LAB}} \|_2);$
return $A, B;$

Algorithm 10: Color characterize image

1. function characterize_color_image ($I_{\text{Raw}}, B_{\text{Raw}}, W_{\text{Raw}}, A, B$):
   Input: Raw color image I_{raw} to be color characterized, raw color image B_{raw} taken without light (camera cover closed), raw color image W_{raw} taken of a uniform white patch, color characterization matrices A and B
    a. **Output:** Characterized color image I_{col}

2. $I_{\text{RGB}} = \text{demosaic}(I_{\text{raw}}, \text{'GRBG'});$
3. $I_{\text{RGB}} = \text{non-local\_means}(I_{\text{RGB}}, 15);$
4. $I_{\text{RGB}} = \text{preprocess\_color\_image}(I_{\text{RGB}}, B_{\text{raw}}, W_{\text{raw}});$
5. **for** x, y **in** $\{1, \dots, w\} \times \{1, \dots, h\}$:
6. $\quad x_{\text{XYZ}} = I_{\text{RGB}}[x, y]\, A;$
7. $\quad x_{\text{Lab}} = \text{CIELAB}(x_{\text{XYZ}}, \text{D50}, 2°);$
8. $\quad I_{\text{Lab}}[x, y] = x_{\text{Lab}}\, B;$
9. $I_{\text{Lab}} = \text{non-local\_means}(I_{\text{Lab}}, 1.5);$
   # Compute specular highlight mask from oversaturation and median lightness
10. $I_{\text{Median}} = \text{median\_filter}(I_{\text{Lab}}, 5, 20);$
11. $I_{\text{Median}} = \text{median\_filter}(I_{\text{Median}}, 3, 20);$
12. **for** x, y **in** $\{1, \dots, w\} \times \{1, \dots, h\}$:
13. $\quad M_H[x, y] = (I_{\text{raw}}[x, y] \geq (2^{12} - 1)) \lor (|I_{\text{Median}}[x, y][1] - I_{\text{Lab}}[x, y][1]| > 10);$
14. $M_H = \text{dilate}(M_H, 5);$
15. **return** $I_{\text{Lab}}, M_H;$

Algorithm 11: Iris segmentation

<u>function segment_iris</u> $(I_{\text{clean}}, B_{\text{raw}}, W_{\text{raw}})$:

Input: Color image I_{RGB} to be pre-processed, raw color image B_{raw} taken without light (camera cover closed), raw color image W_{raw} taken of a uniform white patch

Output: Center coordinates x, y and major and minor radius r_+, r_{minor} and angle α_+ of ellipse fitted to iris

$w, h = 1280, 1024;$

# Compute difference between lightness and chroma

for x, y **in** $\{1, \dots, w\} \times \{1, \dots, h\}$:

  $x_{\text{LCh}} = \text{CIELCh}(I_{\text{clean}}[x, y], \text{D50}, 2°);$

  $I_{\text{enh}}[x, y] = \max(0, x_{\text{LCh}}[1] - x_{\text{LCh}}[2]);$

# Compute center and radius of iris with multi-scale Daugman algorithm

$x, y, r = \text{daugman}(I_{\text{enh}}, (0.25, 0.5, 1.0));$

# Refine circle segmentation to ellipsis by variation of angles and eccentricity at constant area

$A = \pi r^2;$

$\alpha_+, r_+ = 0, r;$

$i_{\max} = -\infty;$

for α **in** $\{0, 5, 10, \dots, 180\}$:

  for r_{major} **in** $\{r, \dots, r + 20\}$:

    $r_{\text{minor}} = A / (\pi\, r_{\text{major}});$

    $c = \sqrt{r_{\text{major}}^2 - r_{\text{minor}}^2};$

    $f_1 = \left(c \cos(\alpha), c \sin(\alpha)\right)^T;$

    $f_2 = \left(-c \cos(\alpha), -c \sin(\alpha)\right)^T;$

    $i_I, i_O = 0,0;$

    for $p = (x, y)$ **in** $\{1, \dots, w\} \times \{1, \dots, h\}$:

      if $\|p - f_1\| + \|p - f_2\| > 2r_{\text{major}}$:

        $i_O = i_O + 1;$

      else:

        $i_I = i_I + 1;$

    if $i_O - i_I > i_{\max}$:

      $\alpha_+, r_+, i_{\max} = \alpha, r_{\text{major}}, i_O - i_I;$

$r_{\text{minor}} = A / (\pi\, r_+);$

return $x, y, r_+, r_{\text{minor}}, \alpha_+;$
