# Supplementary material for: Automatic data-driven design and 3D printing of custom ocular prostheses
Source: Nat Commun. 2024 Feb 27;15:1360. doi: 10.1038/s41467-024-45345-5 (PMC10899237; doi:10.1038/s41467-024-45345-5)
Supplement: Supplementary file 3 — Reporting Summary [file 41467_2024_45345_MOESM3_ESM.pdf]

nature portfolio

Corresponding author(s): Johann Reinhard

Last updated by author(s): Jan 3, 2024

Reporting Summary

Nature Portfolio wishes to improve the reproducibility of the work that we publish. This form provides structure for consistency and transparency in reporting. For further information on Nature Portfolio policies, see our Editorial Policies and the Editorial Policy Checklist.

Statistics

For all statistical analyses, confirm that the following items are present in the figure legend, table legend, main text, or Methods section.

n/a	Confirmed	
☐	☒	The exact sample size (n) for each experimental group/condition, given as a discrete number and unit of measurement
☐	☒	A statement on whether measurements were taken from distinct samples or whether the same sample was measured repeatedly
☒	☐	The statistical test(s) used AND whether they are one- or two-sided<br>*Only common tests should be described solely by name; describe more complex techniques in the Methods section.*
☒	☐	A description of all covariates tested
☒	☐	A description of any assumptions or corrections, such as tests of normality and adjustment for multiple comparisons
☐	☒	A full description of the statistical parameters including central tendency (e.g. means) or other basic estimates (e.g. regression coefficient) AND variation (e.g. standard deviation) or associated estimates of uncertainty (e.g. confidence intervals)
☒	☐	For null hypothesis testing, the test statistic (e.g. F, t, r) with confidence intervals, effect sizes, degrees of freedom and P value noted<br>*Give P values as exact values whenever suitable.*
☒	☐	For Bayesian analysis, information on the choice of priors and Markov chain Monte Carlo settings
☒	☐	For hierarchical and complex designs, identification of the appropriate level for tests and full reporting of outcomes
☒	☐	Estimates of effect sizes (e.g. Cohen's d, Pearson's r), indicating how they were calculated

Our web collection on statistics for biologists contains articles on many of the points above.

Software and code

Policy information about availability of computer code

Data collection	Software and code for the collection of data:<br>The 3D scans of the prostheses were captured with a MEDIT T500 Dental 3D scanner and exported using the companion software provided by the manufacturer. MeshLab (v2020.12) was used to annotate the 3D scans. Patient data was captured with a customized Tomey Casia 2 OCT device that includes a color camera, the companion Tomey Casia software was modified by the manufacturer to provide the raw output of the color camera and the raw OCT images. Microsoft Excel was used to digitally collect the grades assigned by the ocularist.
Data analysis	Software and code for the analysis of data:<br>The creation and analysis of the shape model as well as the computation and analysis of the color characterization were performed using the colormath (3.0.0), matplotlib (3.7.0), numpy (1.24.4), and scikit-learn (1.1.1) Python packages. The proprietary data-driven design software (Cuttlefish:Eye v1.2), described in the publication, was used to process the patient data. It uses OpenCV (4.3.0) for image processing and Eigen (3.3.1) for numerical minimizations. The source code is not available due to licensing contracts but the essential algorithms are provided as pseudo-code and compiled binaries are available from the corresponding author upon reasonable request.

For manuscripts utilizing custom algorithms or software that are central to the research but not yet described in published literature, software must be made available to editors and reviewers. We strongly encourage code deposition in a community repository (e.g. GitHub). See the Nature Portfolio guidelines for submitting code & software for further information.

Data

Policy information about availability of data

All manuscripts must include a data availability statement. This statement should provide the following information, where applicable:

- Accession codes, unique identifiers, or web links for publicly available datasets
- A description of any restrictions on data availability
- For clinical datasets or third party data, please ensure that the statement adheres to our policy

The data of 173 3D-scans of hand-made ocular prostheses, that was used to compute the statistical shape model, is property of Ocupeye Ltd. and available from the corresponding author on request for non-commercial use.
The patient image data used in this study is protected and not available for due to data protection laws. An alternative example or synthetic input data set as well as an accompanying output 3D model is property of Ocupeye Ltd. and available from the corresponding author on request for non-commercial use. Please direct any inquiry with a description of the intended use to johann.reinhard@igd.fraunhofer.de who will acknowledge the request within one week. Sharing of example biometric image data will require a contractual agreement that addresses GDPR compliance.

Research involving human participants, their data, or biological material

Policy information about studies with human participants or human data. See also policy information about sex, gender (identity/presentation), and sexual orientation and race, ethnicity and racism.

Reporting on sex and gender	Information about sex and gender was not collected but inferred from the images. The findings apply to any sex and all gender.
Reporting on race, ethnicity, or other socially relevant groupings	Information has not been collected.
Population characteristics	The reported patients were regular patients of Moorfields Eye Hospital that have lost one of their eyes and required standard clinical care for an ocular prosthesis. All patients were adults, i.e. more than 18 years old, however their age was not recorded. The patients had to be eligible for the procedure, the conditions were similar as for the clinical study NCT05093348. Simply speaking they must be able to keep their eye open and focused on one point for at least 3 seconds. Some patients with conditions such as nystagmus or strabismus cannot be imaged with the OCT device and as such were a priori excluded.<br>Patients that appeared to be eligible were asked if they would be interested in receiving a 3D printed prosthesis, knowing that they would receive a traditional; manually made prosthesis if the 3D printed prosthesis was not satisfactory. Patients gave written informed consent about receiving an 3D printed prosthesis.
Recruitment	In order to find 10 patients that were willing to be shown in this publication starting from 03/27/2023 all patients that opted for the 3D printed artificial eye were asked during their supply visit for explicit written consent to be shown in a scientific publication. This recruitment was stopped on 4/18/2023 after 15 patients were asked of which 11 agreed. The 4 patients that did not provide consent were still supplied with their prosthesis and remarked satisfaction with the result, their assessment by the ocularist was not graded worse than the 11 others.<br>Furthermore two previously supplied patients were included, patient 1 was supplied already on 3/13/2023 and gave consent on 4/11/2023, patient 2 was supplied already 2/10/2023 and gave consent already on this date.<br><br>The quality of the supplied prosthesis and the comfort of the patient with their own appearance could have biased the willingness to consent and thus the results for this selection. Given the patients that declined to give consent to be shown in this submission praised the quality in the assessment indicates that the quality of the prosthesis did not determine patients decision to give consent.<br><br>We completed a simple statistical analysis for the patients that did not consent or were excluded later, see below in "Exclusion", that did not demonstrate a statistically significant difference. Because of incomplete reports and changes in the software version a rigorous analysis for the over 100 patients that have been supplied could not be performed, however we could not see a clinical difference of the results compared to the recruited group. The recruited samples were rather representative, including patients that required an recreated instead of predicted shape and patients that had issues with motility and comfort. Note that the NCT05093348 clinical trial will provide a full statistical analysis for 40 different patients.
Ethics oversight	IRB approval was obtained from the Medical Devices and New Technology Committee of Moorfields Eye Hospital and was registered by the Audit Department, number CA23/RE/960.

Note that full information on the approval of the study protocol must also be provided in the manuscript.

Field-specific reporting

Please select the one below that is the best fit for your research. If you are not sure, read the appropriate sections before making your selection.

☒ Life sciences ☐ Behavioural & social sciences ☐ Ecological, evolutionary & environmental sciences

For a reference copy of the document with all sections, see nature.com/documents/nr-reporting-summary-flat.pdf

Life sciences study design

All studies must disclose on these points even when the disclosure is negative.

Sample size	A sample size of 10 was chosen such to be sufficient to demonstrate the feasibility in a larger scale while not diminishing the long term results of the clinical trial NCT05093348 that includes also the comparison with manually made prostheses. No explicit calculations to determine a sample size were done by us, but we believe that a sample size of 10 is sufficient to demonstrate the viability of our method since the certification of the software as a medical product was done with a similar sample size.
Data exclusions	Of the 13 patients that were recruited 3 were excluded for the following reasons:<br>- One of them was a participant of the clinical trial NCT05093348, it was agreed that these patients will not be shown in this publication.<br>- For one patient an incomplete cosmesis assessment was performed because the adjusted shape turned out to be not optimal during the assessment. The patient was supplied with a fitting prosthesis later on 5/19/2023 after the data analysis was concluded, the report was not completed during that visit.<br>- For one patient the pictures after the adjustment and assessment were taken from too far away, resulting in an image quality and resolution which was insufficient for a publication. Since we intented to visualize the quality of the results for the patients in this publication via the pictures and given that we agreed to show only 10 patients we decided to not include this patient.<br><br>A simple statistical analysis using two-sided Welch-tests with alpha 0.05 indicated that the results for 5 patients with completed assessments not shown (3 due to lack of consent and 2 described above) indicated that the results could belong to the same population.<br>We can provide these reports for these patients on request of the editors.
Replication	We tested that the software produced the same output for the same input data. We tested for one patient in this study that two OCT scans of the same patient give similar shape results. We did not test whether the ocularist could make the same adjustments twice.
Randomization	Since this study only intends to demonstrate that the process for digitally, data-driven designed and 3D-printed prostheses produces useful ocular prosthetics that can be supplied to patients in standard clinical care, without rigorous comparison to traditionally manufactured or other ocular prostheses, no randomization was necessary.
Blinding	Blinding was not desired, since the purpose of this study was to evaluate the quality of the digital prostheses is good enough to supply them to patients and not to compare them with a hand-made prosthesis (which is done in the NCT05093348 clinical trial).

Reporting for specific materials, systems and methods

We require information from authors about some types of materials, experimental systems and methods used in many studies. Here, indicate whether each material, system or method listed is relevant to your study. If you are not sure if a list item applies to your research, read the appropriate section before selecting a response.

Materials & experimental systems

n/a	Involved in the study	
☒	☐	Antibodies
☒	☐	Eukaryotic cell lines
☒	☐	Palaeontology and archaeology
☒	☐	Animals and other organisms
☐	☒	Clinical data
☒	☐	Dual use research of concern
☒	☐	Plants

Methods

n/a	Involved in the study	
☒	☐	ChIP-seq
☒	☐	Flow cytometry
☒	☐	MRI-based neuroimaging

Clinical data

Policy information about clinical studies

All manuscripts should comply with the ICMJE guidelines for publication of clinical research and a completed CONSORT checklist must be included with all submissions.

Clinical trial registration	Not a clinical trial study but proof of concept.
Study protocol	No study protocol because not a clinical trial study.
Data collection	Patient image data using the OCT device was collected for the reported patients from 11/12/2022 to 2/1/2023 with the exception of patient 1 (9/13/2022) and patient 2 (10/12/2022) that were imaged earlier. The patients were recruited during the supply of the prosthesis between 3/27/2023 and 4/18/2023, again with the exception of patient 1 (2/10/2023) and patient 2 (3/13/2023) which were supplied earlier.<br>The report forms to assess the prostheses were filled out during the supply visits. After the assessment the patients kept the adjusted prosthesis as their new permanent ocular prosthesis.<br><br>Approval to supply to these patients of the Moorfields Eye Hospital was granted by its Medical Devices and New Technology committee and registered by the Audit Department number CA23/RE/960. The committee periodically reviewed reports about the

supply and whether any adverse events had been recorded via patient initiated follow-up.

These assessments were primarily conducted to provide feedback necessary to improve the software and not for the purpose to be considered in a publication. Not for all of the more than 100 patients that have been supplied with a 3D-printed prosthesis the assessments have been conducted or completed.

Outcomes

Prostheses were graded by the ocularist during the fitting and supply of the prosthesis with respect to two aspects.

The first aspect was the shape of the software-generated prosthesis. Here the ocularist rated the usually three shapes on a scale from Excellent, Very Good, Acceptable, to Unacceptable. This rating indicates the effort to adjust each shape to the patients eye socket.

After making the adjustments he rated the second aspect cosmesis based on 18 queries, each again on a scale from Excellent, Very Good, Acceptable, to Unacceptable. These questions relate to motility, cosmesis, fit and function, and comfort, which are also part of the NCT05093348 clinical trial. Note however that for these only qualitative results were obtained, while the NCT05093348 clinical trial also computes quantitative results and covers mucous discharge and benefits of use as well as long term effects and comparison with traditionally manufactured PMMA prostheses, and that the patients of NCT05093348 are different patients than shown here.

Additionally the patient was also asked to rate their satisfaction on a scale from Excellent, Very Good, Acceptable, to Unacceptable.
